# Spectral Analysis of Strontium-Doped Calcium Phosphate/Chitosan Composite Films

**DOI:** 10.3390/polym15214245

**Published:** 2023-10-28

**Authors:** Maria Elena Zarif, Bogdan Bita, Sasa Alexandra Yehia-Alexe, Irina Negut, Andreea Groza

**Affiliations:** 1 National Institute for Lasers, Plasma and Radiation Physics, 077125 Măgurele, Romania; maria.zarif@inflpr.ro (M.E.Z.); bogdan.bita@inflpr.ro (B.B.); sasa.yehia@inflpr.ro (S.A.Y.-A.); negut.irina@inflpr.ro (I.N.); 2 Faculty of Chemical Engineering and Biotechnologies, University Politehnica of Bucharest, 011061 Bucharest, Romania; 3 Faculty of Physics, University of Bucharest, 077125 Măgurele, Romania

**Keywords:** strontium-doped calcium-phosphate, radio-frequency magnetron sputtering deposition, matrix assisted pulsed laser evaporation deposition

## Abstract

Strontium-doped calcium phosphate/chitosan films were synthetized on silicon substrates using the radio-frequency magnetron sputtering technique and the matrix-assisted pulsed laser evaporation technique. The deposition conditions associated with the radio-frequency magnetron sputtering discharge, in particular, include the high temperature at the substrate, which promotes the formation of strontium-doped tetra calcium phosphate layers. The physical and chemical processes associated with the deposition of chitosan on strontium-doped calcium phosphate layers were investigated using Fourier Transform Infrared Spectroscopy, Energy Dispersive X-ray Spectroscopy, and Scanning Electron Microscopy. Mass spectrometry coupled with laser induced ablation of the composite films proved to be a useful tool in the detection of the molecular ions characteristic to chitosan chemical structure.

## 1. Introduction

Calcium phosphates (CaPs) have been intensely studied for their biomedical applications. Examples of CaPs are: dicalcium phosphate anhydride [DCPA; CaHPO_4_; Ca/P = 1], dicalcium phosphate dehydrate [DCPD; CaHPO_4_ × 2 H_2_O; Ca/P = 1], octacalcium phosphate [OCP; Ca_8_(HPO_4_)_2_(PO_4_)_4_ × 5 H_2_O; Ca/P = 1.33], α- and β-tricalcium phosphate [α-TCP and β-TCP; Ca_3_(PO_4_)_2_; Ca/P = 1.5], tetra calcium phosphate [TTCP; Ca_4_(PO_4_)_2_O; Ca/P = 2], and hydroxyapatite [Hap; Ca_10_(PO_4_)_6_(OH)_2_; Ca/P = 1.67]. Among them, HAp is the main mineral phase in solid tissues. Biological HAp is calcium deficient [CDHA; Ca_10-x_(HPO_4_)_x_(PO_4_)_6-x_(OH)_2-x_, 0 ≤ x ≤ 1; Ca/P = 1.5–1.67] and carbonate (CO_3_^2−^) substituted [1]. Several vicarious ions such as Mg^2+^ (0.60–0.72 wt%), Sr^2+^ (0.00–0.05 wt%), Zn^2+^ (0.00–39.00 ppm), Cl^−^ (0.1–0.13), F^−^ (0.03–0.10 wt%), Na^+^ (0.9–1.00 wt%), K^+^(0.03–0.07 wt%), and CO_3_^2−^ (4.8–7.40 wt%) are present in bone apatites [2]. 

Synthetic HAp can be obtained using different methods: dry (solid state and mechanochemical), wet (chemical precipitation, hydrothermal, hydrolysis, sol-gel, emulsion, and sonochemical), and high-temperature (combustion and pyrolysis), or a combination of these. Each synthesis technique has its advantages and disadvantages, which influence the properties of the final product (e.g., morphology, phase composition, crystallinity, mechanical, and biological properties, etc.). The hydrothermal synthesis of HAp implies the use of an aqueous solution containing the calcium and phosphate precursors, which is sealed in a reaction vessel and subjected to a high-temperature and high-pressure treatment [3,4,5]. The microwave assisted hydrothermal synthesis uses microwaves for heating, compensating for the drawback of temperature unevenness characteristic of hydrothermal synthesis [6,7]. 

HAp coatings can be obtained via several deposition techniques such as electrochemical [8,9] and electrophoretic [10] depositions, dip coating [11], biomimetic coating [12], plasma spraying [13], flame spraying [11], electron beam physical vapor deposition [14], pulsed laser deposition [15], chemical vapor deposition [14], or radio frequency magnetron sputtering [16]. The advantages and drawbacks of these methods, including cost-and time-efficiency, uniformity of the layer, layer thickness, the ability to cover complicated shapes, and/or additional pre- and post-treatment requirements, must be considered before choosing a specific coating technique for a desired application [11]. 

HAp can be substituted with anionic and cationic ions. Anionic substitution occurs at the PO_4_^3−^ (e.g., CO_3_^2−^, SiO_4_^4−^, SO_4_^2−^) or OH^−^ (e.g., CO_3_^2−^, Cl^−^, F^−^, Br^−^) sites, while cationic substitution occurs at the Ca^2+^ site (e.g., bivalent cations: Sr^2+^, Mg^2+^, Zn^2+^; monovalent cations: Ag^+^, Na^+^, K^+^) [2,17,18]. 

HAp is a stable calcium phosphate with a Ca/P ratio equal to 1.67. The most common form of HAp is the hexagonal phase in the P6_3_/m space group. The ions in the HAp lattice (Ca^2+^, PO_4_^3−^, and OH^−^) are arranged in two symmetric parts of the unit cell. A unit cell contains 10 Ca^2+^ ions, from which four Ca^2+^ ions type I [Ca(I)—arranged in columns parallel to the OH^−^ channels and the c-axis], six Ca^2+^ type 2 [Ca(II)—arranged in a triangular array], 6 PO_4_^3−^ (tetrahedron) ions, and two OH^−^ ions can be found. The Ca(I) is bonded to nine oxygen atoms (belonging to 6 PO_4_^3−^). Six of these bonds are strong and three are weaker, with a Ca(I)-O bond length of 0.255 nm. The Ca(II) is bonded to seven oxygen atoms (belonging to 5 PO_4_^3−^ and OH^−^), with a Ca(II)-O bond length of 0.245 nm [2,17,18,19,20].

Strontium substitution influences the biological properties of HAp, promoting the osteoblast differentiation and proliferation, the alkaline phosphatase (ALP) activity, and the production of collagen type I, decreasing the osteoclast activity, and improving the antimicrobial activity against *Lactobacillus*, *Staphylococcus aureus*, and *Escherichia coli* [2]. Strontium substitution occurs at both calcium sites, Ca(I) and Ca(II), considering their similar ionic radius: 1.00 Å for Ca^2+^ and 1.18 Å for Sr^2+^. At low Sr concentrations (1.00–3.5 at.%) the Ca(I) substitution is favored, while at higher concentrations (5 at.%) Ca(II) substitution is preferred [2,21]. 

Radio frequency magnetron sputtering (RF-MS) discharge is a plasma technique used to deposit various materials on different substrates [22]. The plasma processes involve the bombardment of a sputtering target by energetic ions created in the discharge plasma. The process causes the removal of the target atoms or ions which further are deposed on a substrate as a thin film. The secondary electrons emitted from the target play an important role in maintaining the plasma and in the formation of the coating chemical structure [23]. The advantages of this technique are the good adhesion between the substrate and film, the control of the film chemical composition, the control of temperature during the deposition process, and the control of the uniformity and density of the coatings [24]. The coating composition depends on the discharge power, the deposition time, and the thickness of the film [22].

Matrix Assisted Pulsed Laser Evaporation (MAPLE) is a laser assisted deposition technique which involves the evaporation of a frozen target that consists of a delicate compound dissolved in a volatile solvent. The deposition mechanism implies the fact that the solvent initially absorbs the laser energy, thus preventing the molecules of interest from being damaged by the laser radiation. Further, by continuous laser energy absorbing, the solvent evaporates, and the target molecules are transferred to the substrate and placed in the way of molecular movement, forming a nucleation center, thus the thin film is formed layer by layer. Moreover, due to a low adhesion coefficient, the solvent molecules are pumped away by the pumping system [22,25].

Among other compounds of CaPs, TTCP has the highest Ca/P ratio and it was also obtained as coatings from plasma and laser deposition techniques. Its solubility is higher than that of HAp and it is a biodegradable and biocompatible CaP. It can easily hydrolyse to HAp in humid or aqueous environments, which is why it can only be obtained in dry-air or vacuum environments. As a biomaterial, it is usually used as bone cement [26,27,28].

Mass spectrometry (MS) is an analytical method used for the detection of chemical compounds by measuring the intensities of the collected ions as a function of the mass-to-charge ratio [29]. The recording of the mass spectra after the laser induced ablation (LIA-QMS) of a coating layer allows the detection with high sensitivity of its chemical structure.

This work reports on the synthesis of strontium-doped calcium phosphate (CaPSr)/chitosan films by coupling a plasma deposition technique and a laser evaporation technique. We characterized the films using the following spectral methods: Fourier Transform Infrared Spectroscopy, Energy Dispersive X-ray Spectroscopy, and LIA-QMS, which reveal the processes associated to the chemical and physical structure of the composite layers as function of the deposition conditions. The morphologies of layers were investigated using Scanning Electron Microscopy (SEM).

## 2. Materials and Methods

### 2.1. Materials

Si samples (10 × 10 mm^2^), with a thickness of 2 mm, were used as substrates.

Calcium nitrate tetrahydrate [Ca(NO_3_)_2_·4H_2_O; Sigma-Aldrich; CAS number: 13477-34-4; Molecular weight: 236.15 g/mol], diammonium hydrogen phosphate [(NH_4_)_2_HPO_4_; Fulka; CAS number: 7783-28-0; Molecular weight: 132.06 g/mol], and strontium nitrate [Sr(NO)_3_; 211.63 g/mol] were used as precursors for Ca^2+^, PO_4_^3−^, and Sr^2+^, respectively. Ammonium hydroxide [NH_4_OH; 35.04 g/mol] was used to adjust the pH of the solution. 

**Figure 1:**
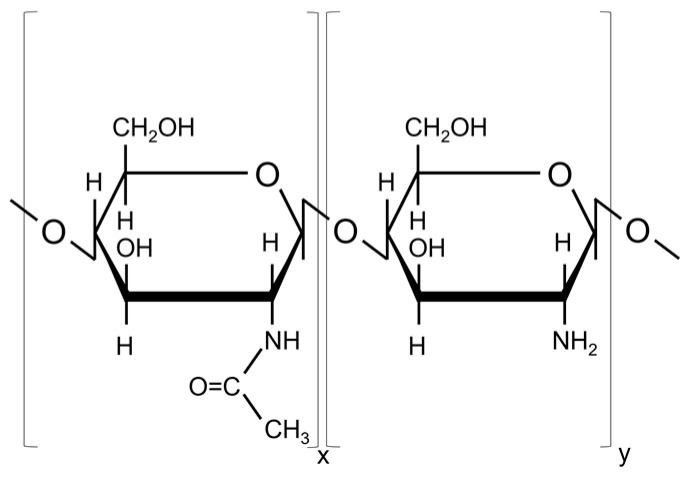
Chemical structure of chitosan (N-acetyl glucosamine and glucosamine units).

Low molecular weight chitosan (C_12_H_24_N_2_O_9_; Sigma-Aldrich, St. Louis, MO, USA; CAS number: 9012-76-4; Molecular weight: 50,000–190,000 Da; Source: shrimp shells; Deacetylation degree: 75–85%) was used to prepare the targets for the MAPLE deposition. The chemical structure is presented in Figure 1.

### 2.2. Synthesis Technique

#### Microwave-Assisted Hydrothermal Synthesis of Sr-Doped Hydroxyapatite

The 3% strontium-doped hydroxyapatite (HApSr; theoretical formula: Ca_9.7_Sr_0.3_(PO_4_)_6_(OH)_2_) was synthetized using microwave-assisted hydrothermal synthesis. Two aqueous solutions of Ca(NO_3_)_2_ + Sr(NO_3_)_2_ and (NH_4_)_2_HPO_4_, respectively, were obtained by dissolving, under continuous stirring, the proper amount of the precursors in distilled water in order to keep the Sr/(Ca + Sr) molar ratio equal to 0.03, and the (Ca+ Sr)/P molar ratio equal to 1.67. The (NH_4_)_2_HPO_4_ solution was added dropwise to the Ca(NO_3_)_2_ + Sr(NO_3_)_2_ solution under continuous stirring using a peristaltic pump set to 50 rpm. The pH of the final solution was adjusted to 10 using NH_4_OH. The hydrothermal synthesis was conducted using the Milestone synthWAVE microwave synthesis system. The solution was heated to 200 °C for 15 min, and maintained at this temperature for 30 min, while the pressure was set to 16 bar. After the synthesis, the equipment was cooled and depressurized. The precipitate was washed with distilled water three times using centrifugal equipment (6000 rpm, 10 min) and dried at 50 °C. After drying, the white powder was mechanically pressed and sputtering targets with diameters of 5 cm and thickness of 4 mm were prepared.

### 2.3. Deposition Techniques

#### 2.3.1. Sr-Substituted Calcium Phosphate Deposition by Radio-Frequency Magnetron Sputtering

The CaPSr layers were deposited on Si substrates using the RF-MS technique, with a magnetron plasma source purchased from K.J. Lesker Company (Jefferson Hills, PA, USA). The depositions were conducted in Ar gas flow (6 ml_n_/min), at a working power of 100 W and a working pressure of ~10^−2^ mbar (base pressure ~10^−5^), for 10 h. The distance between the substrate holder and the magnetron head was set to ~8.5 cm. The thickness of the CaPSr layers is about 300 nm and was calculated after the measurement of the deposition rate [22].

The temperature at the substrate during the deposition process was measured using a single ended thermocouple probe (acquired from K.J. Lesker Company) placed on the substrate surface. After a deposition process of 10 h, it reaches the value of about 200 °C from 25 °C room temperature. The increase of the temperature from 25 °C to 200 °C is due to the energy of particles that attain the substrate: atoms sputtered from the target, argon atoms reflected from the target, and secondary electrons from the target [30]. 

A homemade electronically controlled oven was used for additional heat for the substrate holder during RF-MS deposition from room temperature (25 °C) (CaPSr_1) up to 100 °C (CaPSr_2) and 400 °C (CaPSr_3). These values were measured when plasma was stopped. The results show that the temperature of the substrate during the deposition process varies between 225 and 600 °C. The RF-MS experimental setup schematic representation was previously reported in ref. [22].

#### 2.3.2. Chitosan Deposition by Matrix-Assisted Pulsed Laser Evaporation

The chitosan targets were prepared using a 2% chitosan solution. The chitosan powder was mixed with distilled water under continuous magnetic stirring for 6 h. Acetic acid (200 µL) was also added during the stirring period for complete chitosan dissolution. The MAPLE targets were frozen in liquid N_2_. A KrF* excimer laser source (COMPexPro 205 Lambda Physics-Coherent, λ = 248 nm, τFWHM = 25 ns, Coherent, Santa Clara, CA, USA) was used for the MAPLE depositions. The experiments were conducted at room temperature, at a pressure of 0.1 Pa inside the deposition chamber, under a laser fluence of 300 mJ/cm^2^, and a laser repetition rate of 10 Hz. The substrate-to-target distance was set to 5 cm. During the deposition, the target was rotated to avoid drilling and its temperature was lowered by adding liquid N_2_ into the cooling system. The chitosan layers were deposited onto the CaPSr-coated Si substrates. The MAPLE experimental setup schematic representation was previously reported in ref. [22].

### 2.4. Characterization Techniques

The surface morphology was evaluated using Scanning Electron Microscopy (SEM) and the elemental composition using Energy Dispersive X-ray Spectroscopy (EDX) with a ThermoFisher Apreo S Scanning Electron Microscope (1.3 × 10^−3^ Pa and 8 kV, Waltham, MA, USA) and a SiLi EDX detector. 

The Fourier Transform Infrared Spectroscopy (FTIR) was used for analyzing the molecular structure of CaPSr and CaPSr_CS layers. The ATR-FTIR spectra were acquired in the range of 4000–400 cm^−1^, with a 4 cm^−1^ resolution, using a Perkin-Elmer SP 100 FTIR spectrometer. The IR transmission spectra were transformed into absorption spectra using the SPECTRUM software(version 6.3.5.0176), while the curve-fitting of the spectra was performed using the MagicPlotPro software (version 2.9), as described in ref. [31]. 

The chitosan contained in the CaPSr_CS samples produced in different experimental conditions was structurally and chemically evidenced by laser induced ablation (LIA) of the layers. The species expelled from the layers were analyzed using Quadrupole Mass Spectrometry (QMS). Details on the working procedure and set-up are presented in ref. [32]. A Q-switched laser source was used during the experiments. The laser parameters were the following: pulse repetition rate of 1 kHz, 1053 nm wavelength, 10 ns pulse duration, 350 μJ pulse energy, and 31 J/cm^2^ laser fluence in the focal spot on the target surface. The laser beam profile has a Gaussian shape and the experiments were conducted at an ambience temperature of 16 °C [32]. The mass spectrometer (Pfeiffer QMG 220, Pfeiffer Vacuum, Aßlar, Germany) collected the currents of the following ions characteristic to CaPSr, chitosan, and Si substrate: Si^+^ (m/q = 28), Ca^+^ (m/q = 40), P^+^ (m/q = 31), O^+^ (m/q = 16), C^+^ (m/q = 12), N^+^ (m/q = 14), CaOH^+^ (m/q = 57), PO_4_^+^ (m/q = 95), C_6_H_11_^+^ (m/q = 83), NHCOCH_3_^+^ (m/q = 58), C_5_H_5_O^+^ (m/q = 81), Sr^+^ (m/q = 87.6), CaSr^+^ (m/q = 127.6), SrOH^+^ (m/q = 104.6), and C_6_H_11_NO_4_^+^ (m/q = 161), as a function of time. The Multiple Ions Detection (MID) working mode of the mass spectrometer was used to acquire the data (time resolution 5 ms ± 5%). The Sr^+^, CaSr^+^ and C_6_H_11_NO_4_^+^ ions were not identified during the QMS analysis.

## 3. Results

### 3.1. Scanning Electron Microscopy

**Figure 2:**
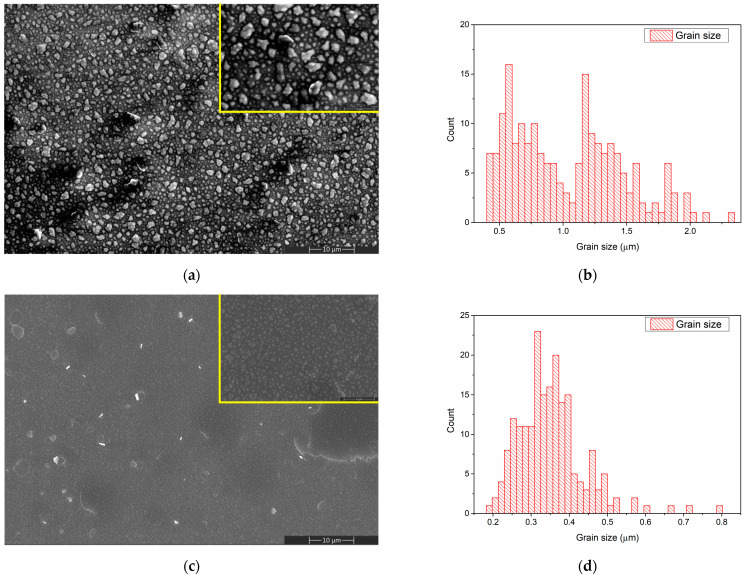

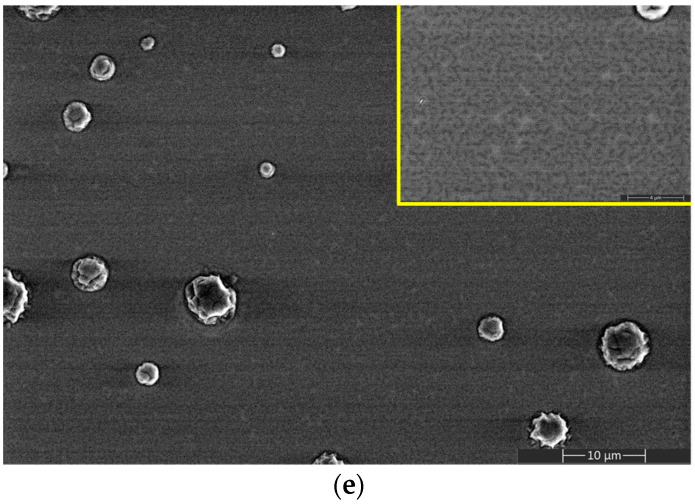
SEM image of CaPSr_1 (**a**), CaPSr_2 (**c**), and CaPSr_3 (**e**) and grain size distribution of CaPSr_1 (**b**) and CaPSr_2 (**d**).

The surface morphology of the CaPSr layers deposited on Si substrates is presented in Figure 2.

By controlling the temperature of the Si substrate during the RF-MS deposition, CaPSr layers with different surface morphologies were obtained (Figure 2). 

A grain-like structure was identified for the CaPSr coating surfaces deposited at room temperature (Figure 2a) and 100 °C (Figure 2c), the grain sizes ranged between 0.4–2.3 μm (Figure 2b), and 0.1–0.8 μm (Figure 2d), respectively. As the temperature of the substrate is oven increased from 25 °C to 100 °C, the grain sizes decrease, and their morphologies change from irregular shapes to quasi-spherical shapes (Figure 2a,c). At the 100 °C substrate temperature, zones where the CaPSr layer partially exfoliates (Figure 2c) were observed. At the substrate temperature of 400 °C, the SEM images (Figure 2e) reveal no grain formation, only microchannels structures, which is possibly due to the coalescence of the grains. As the temperature of the substrate increases, the deposited atoms or molecules gain more thermal energy, migrate to the surface, and rearrange. A similar topography of CaP layers deposited by radio frequency magnetron sputtering technique was also obtained by Bramowicz et al. [33].

Additionally, some spherical structures with diameters ranging between 1 and 7 μm were observed (see Figure 2e). This surface morphology may be caused by the blistering of the layers after the end of the deposition procedure, during the cooling of the substrate holder. The mismatch between the thermal conductivity of the Si substrate and the deposited layers usually produces such effects. 

**Figure 3:**
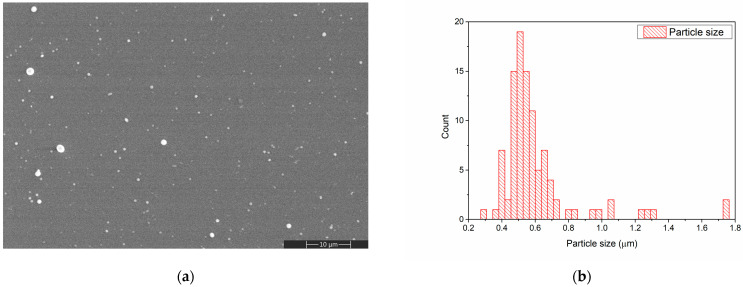
SEM image of CS deposited on Si substrate (**a**) and CS particle size distribution determined from the SEM image (**b**).

The morphology of the chitosan layer deposited by MAPLE on the Si substrate is presented in Figure 3a. During the MAPLE deposition of the chitosan layer some spherical particles, ranging between 0.3 and 0.7 μm in diameter, were formed. Such structures appear due to the splashing of chitosan during laser evaporation [34].

**Figure 4:**
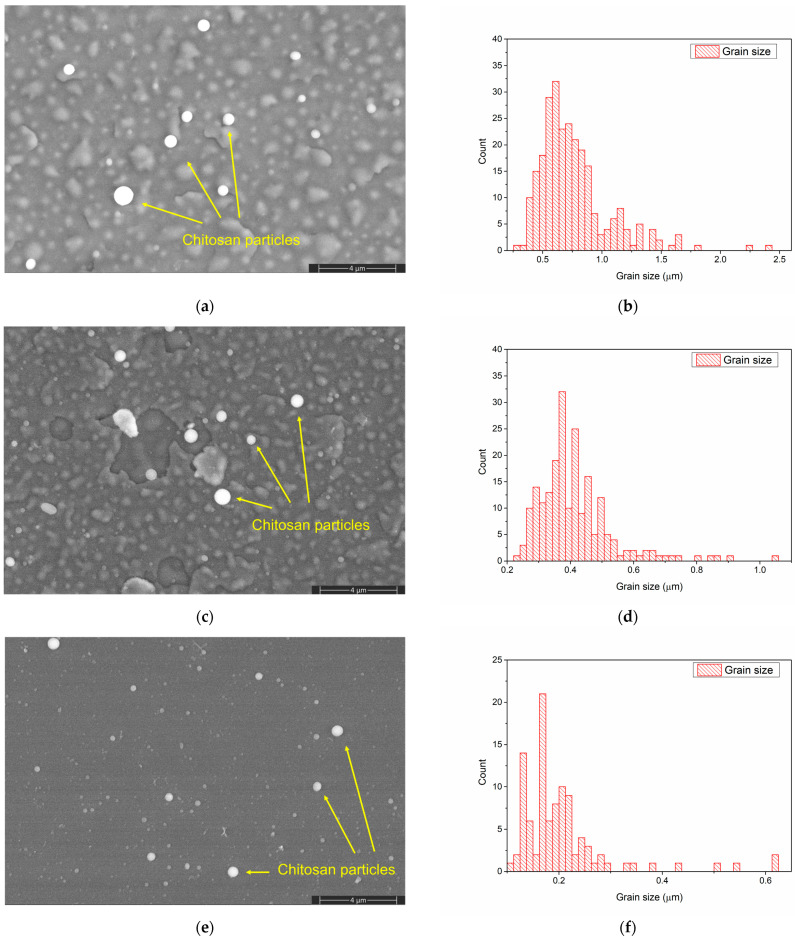
SEM image of CaPSr_CS_1 (**a**), CaPSr_CS_2 (**c**), and CaPSr_CS_3 (**e**) and grain size distribution of CaPSr_CS_1 (**b**), CaPSr_CS_2 (**d**), and CaPSr_CS_3 (**f**).

The morphology of the CaPSr layers (Figure 2a,c,e) is changed after the chitosan deposition (Figure 4a,c,e). The embedding of chitosan during the MAPLE deposition into the CaPSr layers is confirmed by the SEM images. After chitosan deposition onto the CaPSr layers is obtained at substrate temperatures of 25 °C (CaPSr_CS_1) and 100 °C (CaPSr_CS_2), the edges of the grains are smother (Figure 4a,c) compared to those observed for the corresponding CaPSr layers: CaPSr_1 and CaPSr_2 (Figure 2a,c). These findings confirm that chitosan was embedded between the CaPSr grains. Moreover, the changes in the grain size distribution (Figure 2b,d and Figure 4b,d) sustain the above ascertainment. The thicknesses of the layers increased following the chitosan deposition, with lower values for CaPSr_CS_1, ~370 nm, and higher values for CaPSr_CS_2, ~398 nm. These results may indicate a deeper embedding of the chitosan layer into the CaPSr coating deposited without oven-controlled heating of the substrate. The embedding of chitosan into the CaPSr layer deposited at a substrate temperature of 400 °C is evidenced by the SEM images, as the microchannels observed for CaPSr_3 (Figure 2e) were no longer observed for CaPSr_CS_3 (Figure 4e). However, the cross-sectional SEM images (not showed here) evidenced that the chitosan was mainly deposited at the surface as microparticles, leading to a layer thickness of up to ~700 nm. These results are sustained by our previous research [22] in which calcium phosphate/chitosan composite layers were obtained on Ti samples using the RF-MS and MAPLE techniques. As the temperature of the substrate was increased, the CaP layers became more compact and chitosan was mainly deposited at the surface of the samples. Chitosan microparticles were also observed in the SEM images for the CaPSr_CS layer surfaces. 

### 3.2. Energy Dispersive X-ray Spectroscopy

The specific chemical elements in CaPSr (Ca, P, O, Sr), along with Si, characteristic of the substrate, were identified in all the EDX spectra of the CaPSr coatings. The atomic ratios for the HApSr powder obtained through the Microwave-assisted Hydrothermal synthesis, calculated from the EDX measurements, are 1.70 for the (Ca + Sr)/P ratio and 0.024 for the Sr/(Ca + Sr) ratio. The slightly lower Sr/(Ca + Sr) ratio may indicate that part of the Sr ions remained in the solution during the synthesis [35]. 

**Figure 5:**
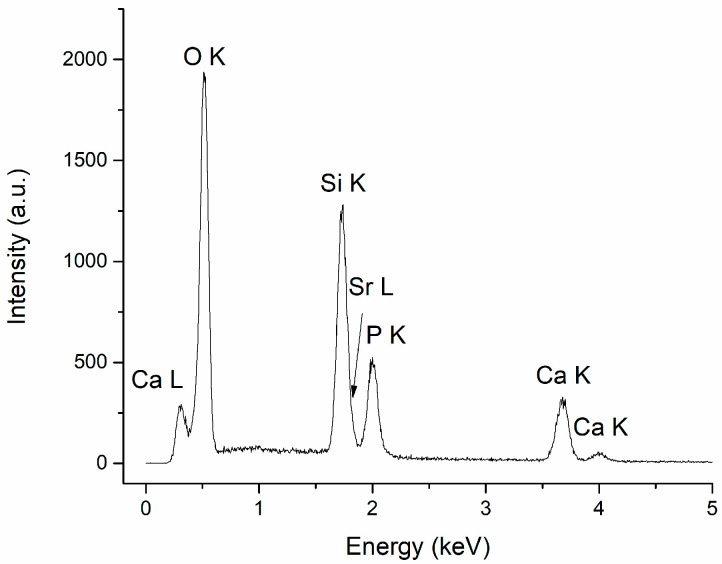
EDX spectrum of CaPSr_1.

The EDX spectrum of the CaPSr_1 coating is presented in Figure 5.

The atomic ratios for (Ca + Sr)/P and Sr/(Ca + Sr) calculated for the CaPSr coatings, following the EDX measurements, are presented in Table 1. The (Ca + Sr)/P ratios are similar for all coatings (~2), regardless of the substrate temperature, as an indication of Sr-doped TTCP. We suppose that the high temperature from the substrate during the deposition process does not allow the incorporation of hydroxyl groups in the apatite structure, which is essential for the recovery of the Sr-doped HAp structure on the substrate. It is known that, during the RF magnetron sputtering deposition, HAp decomposition may appear, leading to TTCP formation, which increases the Ca/P ratio [27]. 

The Sr/(Ca + Sr) ratio has the lowest value for the CaPSr coating deposited without oven heating of the substrate during the RF-MS deposition (CaPSr_1) and the highest value for the CaPSr coating deposited at a substrate temperature of 100 °C. The Sr/(Ca + Sr) atomic ratio calculated for the CaPSr coating deposited without oven heating of the substrate (0.030 ± 0.003) was the closest to the theoretical value of 0.03. 

**Figure 6:**
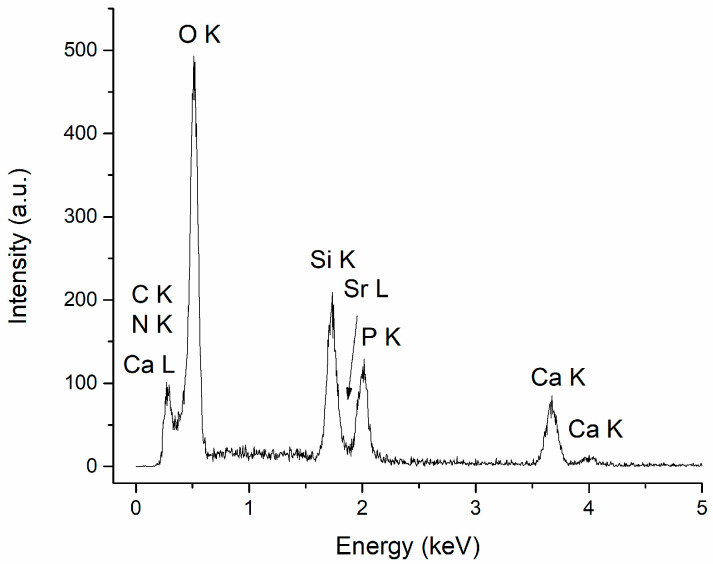
EDX spectrum of CaPSr_CS_1.

The specific chemical elements in CaPSr (Ca, P, O, Sr) and chitosan (C, N), along with Si, characteristic of the substrate, were identified in all the EDX spectra of the CaPSr_CS coatings. The EDX spectrum of the CaPSr_CS_1 is presented in Figure 6. 

The atomic ratios for the CaPSr_CS coatings, calculated from the EDX measurements, are presented in Table 2. The (Ca + Sr)/P ratios for the coatings deposited without oven-controlled heating of the substrate (CaPSr_CS_1) and by oven heating of the substrate at 100 °C (CaPSr_CS_2) are similar to those reported for the CaPSr coatings without CS (CaPSr_1 and CaPSr_2, respectively). For the coating deposited at a substrate temperature of 400 °C, the (Ca + Sr)/P ratio decreased from 2.09 ± 0.05 for the CaPSr coating to 1.76 ± 0.04 for the CaPSr_CS coating. 

### 3.3. Fourier Transform Infrared Spectroscopy

The FTIR analysis was required in order to identify the chemical groups characteristic of CaPSr and CaPSr_CS. The main functional group that manifests vibrations specific to the doped or undoped CaP is PO_4_^3−^. In CaP, the P-O bonds’ vibration frequencies in the PO_4_^3−^ group are identified as: 1120–1000 cm^−1^ (ν_3_), 960 cm^−1^ (ν_1_), 600–550 cm^−1^ (ν_4_), and 470 cm^−1^ (ν_2_) [1,36]. There are several papers that have previously indicated that the doping of calcium phosphate compounds or their formation in multiple physical states (α-TCP and β-TCP, Ca_3_(PO_4_)_2_, Ca/P = 1.5; TTCP, Ca_4_(PO_4_)_2_O, Ca/P = 2; CaHPO_4_, Ca/P = 1; CaHPO_4_ × 2 H_2_O, Ca/P = 1; Ca_8_(HPO_4_)_2_(PO_4_)_4_ × 5 H_2_O, Ca/P = 1.33) leads to the occurrence of P-O bond vibrations at certain wavenumbers. 

**Figure 7:**
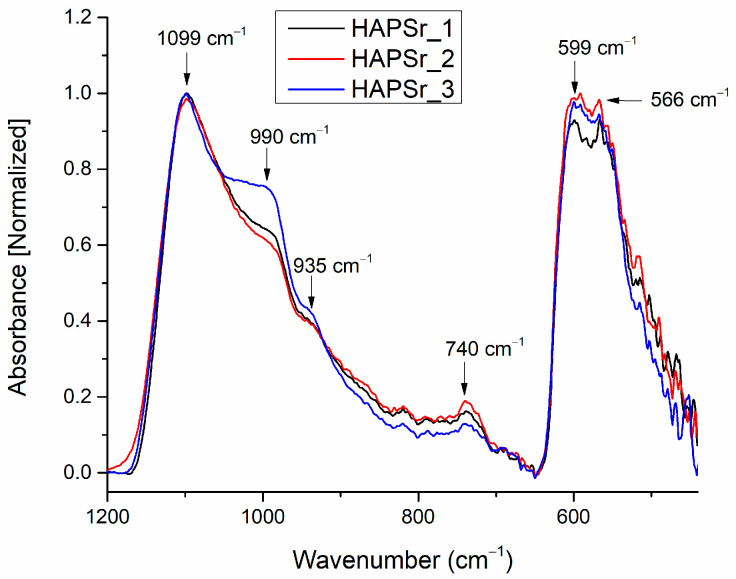
FTIR spectra of the CaPSr coatings deposited on Si substrates at different substrate temperatures (CaPSr_1, CaPSr_2, and CaPSr_3).

Figure 7 presents the FTIR spectra of the CaPSr coatings deposited on Si at different substrate temperatures in the wavenumber range of 1200–500 cm^−1^. We did not observe absorption bands at 3570 cm^−1^ (characteristic of the stretching mode of the OH group in the lattice water), and 1640 cm^−1^ (characteristic of the bending mode of the OH group in the water adsorbed on the film surface).

In the wavenumber range 1200–800 cm^−1^ (see Figure 7), IR bands were identified at the following wavenumbers: 1099 and 990 cm^−1^, characteristic of the asymmetric stretching of the phosphate ion (ν_3_ PO_4_^3−^) [27,28,35]; 935 cm^−1^_,_ characteristic for the symmetric stretching (ν_1_) of PO_4_^3−^ [22]; and 740 cm^−1^, characteristic of P_2_O_7_^4−^, possible formed during the plasma deposition [1]. Paper [28] reports findings of the symmetric stretching of PO_4_^3−^ ions at 941 cm^−1^. In our previous paper [37], which reported the generation of CaPCS layers with magnetron sputtering technique from a CaPCS target, the (ν_1_) vibrational mode of the phosphate ion was identified at 940 cm^−1^. Therefore, we suppose that the doping of CaP with Sr or the deposition conditions of the layers leads to shifts in the wavenumber of the IR bands specific to CaPSr samples. 

In the wavenumber range 650–400 cm^−1^ (see Figure 1), two absorption bands, characteristic of the bending mode of PO_4_^3−^ ions in apatite structure, were identified: at 599 cm^−1^ and 566 cm^−1^ [21,35,38,39]. There are no absorption bands at 630 cm^−1^, characteristic of OH groups in HAp structures.

The results presented above indicate that the high temperature from the substrate was conducive to the absence of the OH groups in the CaPSr films. These insights, together with the measured ratio of the (Ca + Sr)/P of ~2, indicate the synthesis of CaPSr layers as Sr-doped TTCP. 

**Figure 8:**
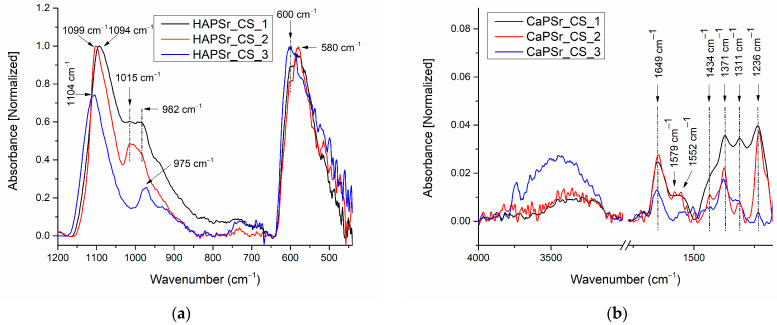
FTIR spectra of the CaPSr_CS composite coatings deposited on Si substrates at different substrate temperatures (CaPSr_CS_1, CaPSr_CS_2, and CaPSr_CS_3) in the wavenumber range 1200–450 cm^−1^ (**a**) and 4000–1200 cm^−1^ (**b**).

The addition of chitosan to the CaPSr samples leads to the modification of the wavenumber positions of the P-O vibrations in PO_4_^3−^ groups. Figure 8 presents the FTIR spectra of the CaPSr_CS samples.

In the wavenumber range 1200–800 cm^−1^, it can be observed that, as the substrate was oven heated from room temperature to 400 °C, the 1094 cm^−1^ IR band (CaPSr_CS_1 sample) was shifted to higher wavenumbers up to 1104 cm^−1^. For the CaPSr_CS_2 sample, this absorption band is at the same wavenumber as in the case of the CaPSr_2 sample (1099 cm^−1^). On the contrary, the IR bands observed in the IR spectra of the CaPSr samples at ~990 cm^−1^, shift to lower wavenumber values: 982 cm^−1^. A peak at 1015 cm^−1^, characteristic of the saccharide structure of chitosan [40], was identified for the CaPSr_CS_1 and CaPSr_CS_2 samples (see Figure 8). 

In the wavenumber range 650–400 cm^−1^, the absorption bands specific to the ν_4_ vibrational mode of PO_4_^3−^ ions are slightly different than those presented in Figure 7. In the case of the CaPSr_CS_1 and CaPSr_CS_2 samples, an absorption band centered at 580 cm^−1^ is observed, while for the CaPSr_CS_3 sample the absorption band appears at 600 cm^−1^.

We suppose that the decrease in the intensity of the 1200–900 cm^−1^ IR band can be attributed to the reduced number of P-O vibrations in the ν_3_ mode, due to chitosan addition (see Figure 2 and Figure 4). Additionally, the position modifications of the IR band, characteristic of the bending modes of PO_4_^3−^ ions in apatite structures, suggest that the physicochemical processes are encountered between the CaPSr layer and the chitosan.

In Figure 8b and Table 3 are presented the IR bands characteristic of chitosan identified in the 4000–1200 cm^−1^ spectral range.

#### 3.3.1. Peak Fitting Analysis of FTIR Spectra of CaPSr Coatings s

A peak fitting analysis was conducted for revealing the molecular structure of CaPSr and the embedding of chitosan into the CaPSr layers. In addiiton, the influence of the substrate temperature on the layers molecular structure was analyzed. 

**Figure 9:**
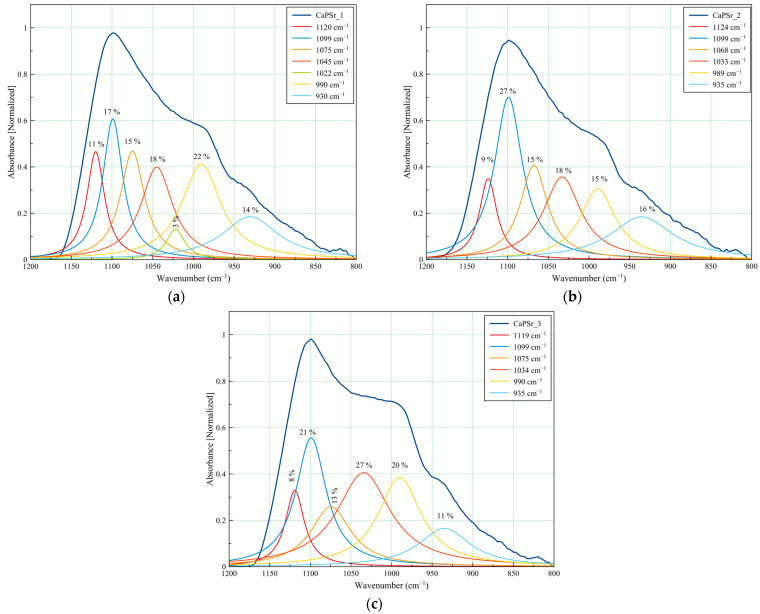
Deconvoluted FTIR spectra of the CaPSr coatings deposited at different substrate temperatures: CaPSr_1 (**a**), CaPSr_2 (**b**), and CaPSr_3 (**c**) in the 1200–800 cm^−1^ wavenumber range.

The deconvolution spectra of the CaPSr coatings are presented in Figure 9 and Table 4.

The CaPSr_1, CaPSr_2, and CaPSr_3 samples have similar peak area percentages (Figure 9) for the absorption bands identified at 1120, 1124, and 1119 cm^−1^, respectively. Previously, Gadaleta et al. assigned the bands at 1127 cm^−1^ to non-stoichiometric apatite [22,47]. 

The absorption band at 1022 cm^−1^ (see Figure 9a) was no longer observed when the substrate was oven heated during the deposition process (see Figure 9b,c). As the substrate temperature was oven increased up to 400 °C, this absorption band seems to have shifted to ~1030 cm^−1^ (see Figure 9b), while the peak area percentage increased from 18 to 27% (Figure 9b,c). The 1030 cm^−1^ IR band was formerly attributed to the formation of the CaPSr structure [28,39]. These results suggest that, as the temperature of the substrate is oven increased, Sr-doped apatite structure formation is promoted.

The absorption bands at ~990 cm^−1^ and ~935 cm^−1^ had no major shifts in the analyzed samples, with peak area percentages ranging between 15–22% and 11–16%, respectively.

**Table 4:** The assignment of FTIR absorption bands for the CaPSr and CaPSr_CS coatings in the wavenumber domain 1200–800 cm^−1^.

Wavenumber (cm^−1^)						FTIR Band Assignment	Ref.
CaPSr_1	CaPSr_2	CaPSr_3	CaPSr_CS_1	CaPSr_CS_2	CaPSr_CS_3		
					1134	Asymmetric stretching of C-O-C	[41,48]
1120	1124	1119	1111	1108	1119	P-O vibration in non-apatite phosphate structure	[47]
1099,1075, 1045, 1022	1099,1068, 1033	1099, 1075,1034	1094,1073, 1047	1099, 1080,1055,	1104, 1082, 1055	ν_3_ PO_4_^3−^	[21,35,38,39,49,50,51]
990	989	990	982	982	976	ν_3_ PO_4_^3−^ in TTCP	[27,28]
		52	1015	1015		polysaccharide structure of CS	[40]
930	935	935	934	929	929	ν_1_ PO_4_^3−^	[22,37]

#### 3.3.2. Peak Fitting Analysis of FTIR Spectra of CaPSr_CS Coatings 

**Figure 10:**
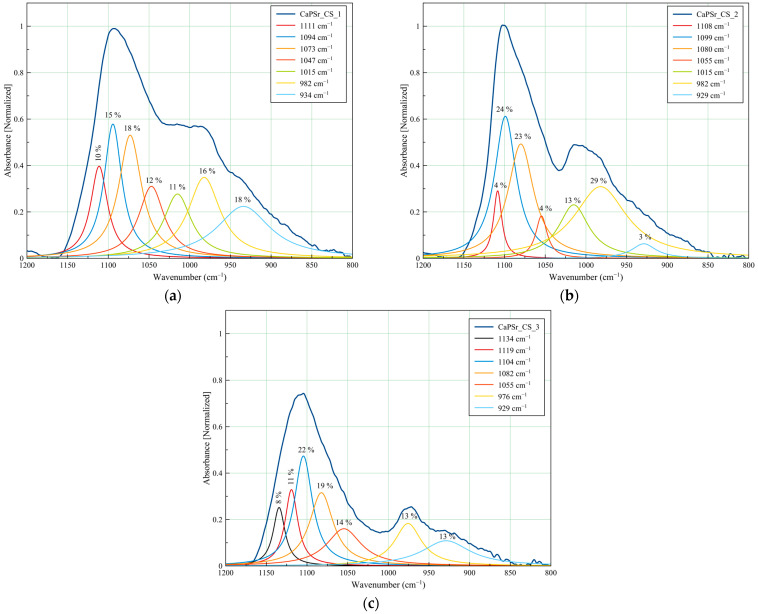
Deconvoluted FTIR spectra of the CaPSr coatings deposited at different substrate temperatures: CaPSr_CS_1 (**a**), CaPSr_CS_2 (**b**), and CaPSr_CS_3 (**c**) in the 1200–800 cm^−1^ wavenumber range.

The peak fitting analysis of the CaPSr_CS samples FTIR spectra are presented in Figure 10 and Table 4. Slight modifications of the deconvoluted IR band positions, as functions of the oven-controlled temperature at the substrate during the deposition process of the CaPSr layers, can be observed. The addition of chitosan on their surface is conducive to these modifications due to some physicochemical interactions. In Figure 10a,b the deconvoluted IR band from 1015 cm^−1^, specific to chitosan structure, is evidenced. Although this band did not appear in the deconvoluted spectrum of the CaPSr_CS_3 sample, a new deconvolution band was observed at 1134 cm^−1^ which that could be attributed to the asymmetric stretching of C-O-C bonds of the chitosan structure [41,48].

### 3.4. QMS Monitoring of the Species Released from the CaPSr_CS Coatings during LIA Process

**Figure 11:**
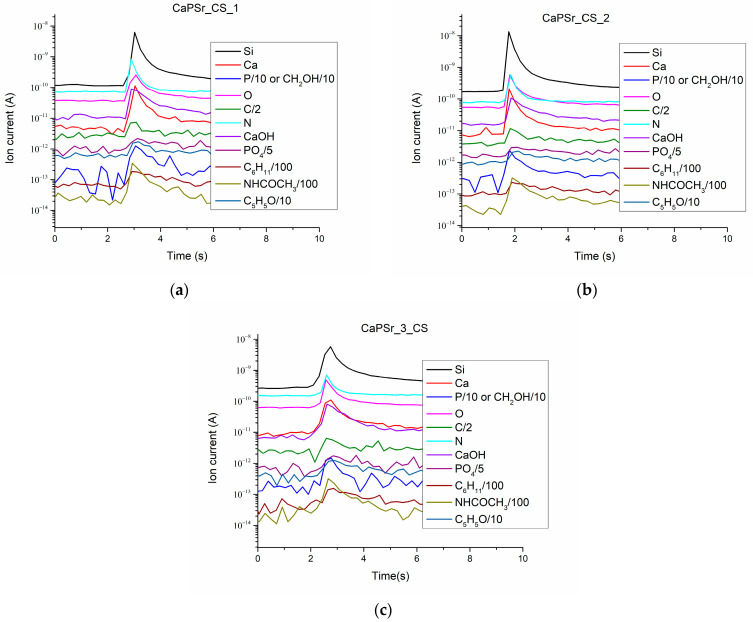
Ion current as a function of time for the species released from the CaPSr_CS coatings during LIA at nominal laser pulse energy: CaPSr_1_CS (**a**), CaPSr_2_CS (**b**), and CaPSr_3_CS (**c**).

The ion currents of the species released during the laser–sample interaction from the CaPSr_CS layers deposited on Si substrates are presented in Figure 11. 

The collected Ca^+^ (m/q = 40), CaOH^+^ (m/q = 57), P^+^ (m/q = 31), and PO_4_^+^ (m/q = 95) ion currents are characteristic of the apatite structure of the layers. These positive molecular ions specific to calcium phosphate were previous identified by time-of-flight secondary ion mass spectrometry [52,53,54,55,56,57].

The presence of chitosan in the CaPSr_CS coatings is highlighted mainly by the detection of the following molecular ions: C_6_H_11_^+^ (m/q = 83), NHCOCH_3_^+^ (m/q = 58), C_5_H_5_O (m/q = 81). The detection of NHCOCH_3_^+^ (m/q = 58) is due to the N-acetyl glucosamine unit of the chitosan structure.

The C^+^, N^+^, O^+^ ions were also identified. The signal of the Si^+^ ion current confirms that, during the laser ablation of the layers, the substrate is attained. This means that chitosan is embedded in the CaPSr layers, except, perhaps the microparticles formed on their surfaces. Moreover, the simultaneous detection of ions characteristic both of CaPs and chitosan and the SEM analysis of the layer morphologies sustain the above ascertainment.

## 4. Conclusions

Strontium-doped calcium phosphate/chitosan composite layers were synthetized using plasma and laser techniques, namely radio-frequency magnetron sputtering and matrix-assisted pulsed laser evaporation. 

The formation of strontium-doped tetra calcium phosphate layers was possible by tuning the temperature of the substrate during the magnetron sputtering deposition processes. The morphology of the layer surfaces evolves from grain structures to microchannels structures, with increases in the temperature of the substrate.

The FTIR and EDX spectral analysis indicated the absence of OH groups from apatite structure (no molecular bands at 3570 and 630 cm^−1^) and the (Ca+Sr)/P atomic ratio of ~2, that are characteristic of tetra calcium phosphate compounds. The further deposition of chitosan on strontium-doped calcium phosphate layers does not change these data. 

The presence of chitosan in the layers was highlighted by SEM, FTIR spectroscopy, and LIA—QMS analysis. All the IR bands characteristic of chitosan were revealed. The collected mass spectra showed C_6_H_11_^+^ (m/q = 83), NHCOCH_3_^+^ (m/q = 58), and C_5_H_5_O^+^ (m/q = 81) ions, specific to the chitosan chemical structure.

## Figures and Tables

**Table 1:** EDX (Ca + Sr)/P and Sr/(Ca + Sr) atomic ratios for the CaPSr coatings.

Atomic Ratio	CaPSr_1	CaPSr_2	CaPSr_3
(Ca + Sr)/P	2.01 ± 0.05	2.04 ± 0.04	2.09 ± 0.05
Sr/(Ca + Sr)	0.030 ± 0.003	0.045 ± 0.003	0.035 ± 0.005

**Table 2:** EDX (Ca + Sr)/P and Sr/(Ca + Sr) atomic ratios for the CaPSr_CS coatings.

Atomic Ratio	CaPSr_1_CS	CaPSr_2_CS	CaPSr_3_CS
(Ca + Sr)/P	2.02 ± 0.08	2.10 ± 0.15	1.76 ± 0.04
Sr/(Ca + Sr)	0.028 ± 0.003	0.051 ± 0.011	0.046 ± 0.009

**Table 3:** The assignment of FTIR absorption bands for the CaPSr_CS coatings in the wavenumber domains: 4000–1200 cm^−1^.

Wavenumber (cm^−1^)			FTIR Band Assignment	Ref.
CaPSr_CS_1	CaPSr_CS_2	CaPSr_CS_3		
3590–3085	3590–3085	3700–3085	polymeric O-H stretch or N-H	[41]
1649	1646	1653	stretching of (-C=O-) of amide I group/N-H stretching of amide I	[34,42]/[43]
	1579		N-H straining vibrations of -NH_2_	[43,44]
1556	1552	1556	bending of N-H in amide II	[41]
1434	1434	1431	asymmetric bending of C-H in (-CH_3_)	[41]
1371	1376	1378	Symmetric bending of C-H in (-CH_3_)	[34,41,45]
1311	1314	1311	stretching of (-CH_3_) of amide III groups	[34]
1236	1231	1234	-NHCO-	[46]

## Data Availability

The data presented in this study are available on request from the corresponding author.
